# Fat Grafting Breast Augmentation Mammaplasty

**Published:** 2013-08-08

**Authors:** Karan Chopra, Kashyap K. Tadisina, Nikki Chopra, Marwan R. Khalifeh

**Affiliations:** Department of Plastic and Reconstructive Surgery, The Johns Hopkins Hospital, Johns Hopkins University School of Medicine, Baltimore, Md

**Keywords:** fat grafting, breast augmentation, aesthetic surgery

**Figure:**
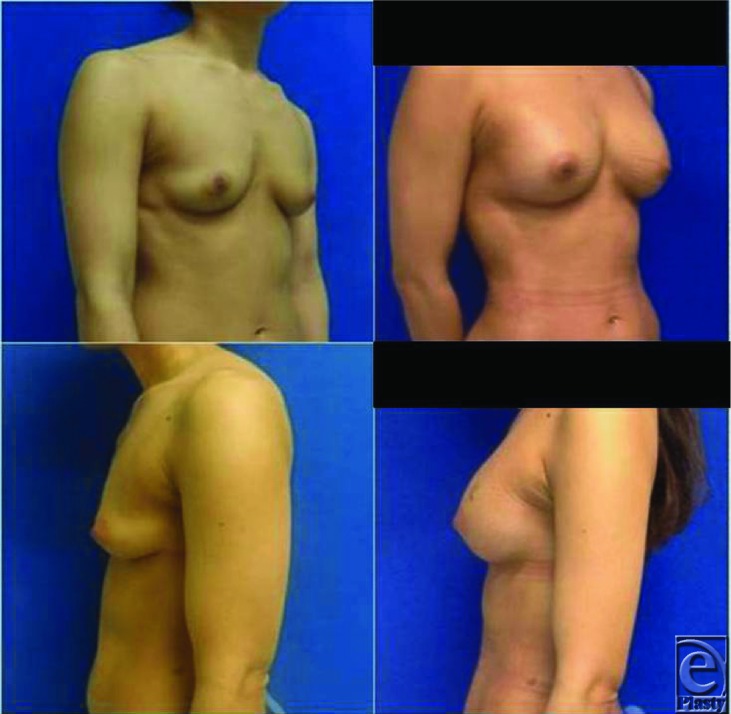


## DESCRIPTION

A middle-aged woman presented to the plastic surgery clinic for breast augmentation. Fat grafting augmentation mammaplasty was performed successfully.

## QUESTIONS

1. **Describe the history of autologous fat grafting in breast augmentation.**
2. **When is fat grafting used and what is the appropriate technique?**
3. **What are the main advantages of the fat grafting technique?**
4. **What are complications associated with fat grafting?**

## DISCUSSION

Fat grafting had been employed as a method of breast augmentation as early as 1895 when Czerny first transplanted a lipoma from the back to the breast for a mastectomy patient. Fat grafting involves the harvest of donor adipose tissue, most commonly from the buttocks or lower abdomen, and transplanting it to the patient's breast tissue. It is commonly used in the reconstructive setting as an adjunct to flap or implant breast augmentation^1^ to augment areas of deflation such as the upper pole of the breast or to decrease contour irregularities. Fat grafting is also useful as a primary technique for small-volume augmentations^2^ or reconstructions (ie, postlumpectomy reconstruction). The technique is not without controversy, originally being deplored as a viable technique for breast augmentation by the American Society of Plastic and Reconstructive Surgeons Ad-Hoc Committee on New Procedures in 1987. The main concern was the potential interference with breast cancer screening. Since then, concurrent with the rise of liposuction techniques for body contouring, fat grafting techniques have become more popular. In 2009, the Fat Grafting Task Force of the American Society of Plastic Surgeons released a study describing the popular use of fat grafting by surgeons and the need for more research into the technique, without discouraging or encouraging its further use in practice. Currently, fat grafting is a highly researched area of breast augmentation, with current techniques exploring the potential benefits of the adipose-derived stem cells present in the graft and other modifications to optimize results and safety.^1^ Further debates about its safety have also created the demand for a national registry to examine risk stratification, standardized protocols, and true assessment of its impact on cancer and screening.^4^

A recent survey found that up to 62% of plastic surgeons use fat grafting as a technique of breast reconstruction and up to 28% use it as an aesthetic technique. In both cases, the preference is for fat grafting to be used as an adjunct technique along with flap or implant use.^1^ Adipose tissue is harvested from the donor site, usually the abdomen or inner thigh and then prepared according to the surgeons' personal preference. From here, the fat is separated into several syringes and directly transferred into the recipient breast under low pressure. Generally, a higher amount of fat is injected than is finally desired to compensate for fat resorption. Commonly, a 140-cc injection may result in approximately 100-cc final graft survival.^5^

The main advantage of fat grafting augmentation is the natural appearance and feel and the lack of any of the risks associated with implanting an alloplastic breast prosthesis. Fat grafting techniques have been used for not only breast augmentation but also body contouring of the face and torso. Adipose tissue is an optimal soft tissue filler material that is natural to a patient's body and is relatively abundant in the body.^6^ When used in conjunction with implants, fat grafting has even been found to soften capsular contracture.^7^ As an all-natural substance, fat grafting gives patients and their surgeons an all-natural option for breast augmentation that does not require the insertion of a foreign body.

Plastic surgeons experienced in this technique report reliable results. Overall, fat grafting for breast augmentation has a steep learning curve and is difficult to master. Like any plastic surgery operation, it is associated with its own set of potential complications. Potential complications include pain in the immediate postoperative period, bleeding, localized infection of the harvest or injection site, fat resorption and necrosis, and suboptimal aesthetic outcome. Furthermore, there is a theoretical risk of fat grafting interfering with breast cancer detection, but preliminary scientific evidence does not support this notion and has found no increased risk of cancer in fat graft augmentation patients.^6^
